# Serum Hepcidin Levels and Reticulocyte Hemoglobin Concentrations as Indicators of the Iron Status of Peritoneal Dialysis Patients

**DOI:** 10.1155/2012/239476

**Published:** 2012-11-01

**Authors:** Aya Eguchi, Takahiro Mochizuki, Misao Tsukada, Koji Kataoka, Yukio Hamaguchi, Shinichiro Oguni, Kosaku Nitta, Ken Tsuchiya

**Affiliations:** ^1^Department of Medicine IV, Tokyo Women's Medical University, Kawada-cho 8-1, Shinjuku, Tokyo 162-8666, Japan; ^2^Department of Nephrology, Kameda Medical Center, Higashi-machi 929, Kamogawa, Chiba 296-8602, Japan; ^3^Immunology & Chemistry Product Engineering, ICH Business Unit, Sysmex Corporation, Wakihama, Kaigandori 1-5-1, Chuou-ku, Koube, Hyogo 651-0073, Japan; ^4^Clinical Development, Technology Development, Sysmex Corporation, Wakihama, Kaigandori 1-5-1, Chuou-ku, Koube, Hyogo 651-0073, Japan

## Abstract

Hepcidin is the key mediator of renal anemia, and reliable measurement of serum hepcidin levels has been made possible by the ProteinChip system. We therefore investigated the iron status and serum hepcidin levels of peritoneal dialysis (PD) patients who had not received frequent doses of an erythrocytosis-stimulating agent (ESA) and had not received iron therapy. In addition to the usual iron parameters, the iron status of erythrocytes can be determined by measuring reticulocyte hemoglobin (RET-He). The mean serum hepcidin level of the PD patients (*n* = 52) was 80.7 ng/mL. Their serum hepcidin levels were significantly positively correlated with their serum ferritin levels and transferrin saturation (TSAT) levels, but no correlations were found between their serum hepcidin levels and RET-He levels, thereby suggesting that hepcidin has no effect on the iron dynamics of reticulocytes. Since low serum levels of CRP and IL-6, biomarkers of inflammation, were not correlated with the serum hepcidin levels, there is likely to be a threshold for induction of hepcidin expression by inflammation.

## 1. Introduction

Anemia is one of the major problems in the management of complications that occurs in peritoneal dialysis (PD) patients who have neither received frequent doses of an erythropoiesis-stimulating agent (ESA) nor received iron therapy. Several factors unique to PD patients, including exposure to PD solution, episodes of peritoneal infection (peritonitis), and biological changes in the peritoneum, in addition to a basic deficiency of intrinsic erythropoietin and dysregulation of iron metabolism, may be involved in the pathogenesis of the anemia. Clinical and subclinical chronic inflammation may contribute to the etiology of the renal anemia that also sometimes develops in PD patients.

Hepcidin expression is stimulated by inflammation and by iron loading, and hepcidin is the key mediator of renal anemia [1]. Human hepcidin is a 25-amino acid peptide synthesized by hepatocytes, and it may be a mediator of innate immunity and the long-sought iron-regulatory hormone [2]. Hepcidin expression is greatly stimulated by inflammation and by iron overload, and hepcidin maintains iron homeostasis. Hepcidin activity is also partially responsible for the iron sequestration seen in the anemia of chronic disease [3], and serum hepcidin levels are elevated in chronic kidney disease (CKD) patients [4]. Reliable serum hepcidin measurements have been made possible by the ProteinChip system [4], but no clear, direct correlations between serum hepcidin levels and iron parameters have been found. In addition to being able to measure the usual iron parameters by the routine methods, it has recently become possible to determine the iron status of erythrocytes by measuring reticulocyte hemoglobin [5, 6]. The conventional method of diagnosing iron deficiency involves measuring serum iron levels, ferritin levels, and transferrin saturation (TSAT) levels, but they are indirect markers. The ideal method of evaluating iron status would be one that directly measures the iron content of erythrocytes, particularly of newly produced erythrocytes. Reticulocyte hemoglobin content (RET-He) can now be measured by a flow cytometric technique [7]. RET-He is a reticulocyte parameter that is thought to reflect hemoglobin synthesis by erythrocytes newly formed in the bone marrow in real time.

In order to clarify the relationship between the iron status and serum hepcidin levels of PD patients, in this study, we investigated the iron status and serum hepcidin levels of PD patients who had neither received frequent doses of ESA nor frequent iron therapy.

## 2. Subjects and Methods

### 2.1. Patients

 The protocol of this study and the informed consent form were approved by the hospital's institutional review board, and the study was carried out according to the principles of *the Declaration of Helsinki*. Informed consent was obtained from all of the subjects. 

Table 1 indicates the partition of the patients. Fifty-two patients who were undergoing PD at Tokyo Women's Medical University or Kameda Medical Hospital were enrolled. Anemia was defined as a Hb concentration <10 g/dL. Iron deficiency was defined as a TSAT level <20% and a serum ferritin level <100 ng/mL. The data were cross-sectionally sampled in the patients. We excluded patients in whom there had been any change in rHuEPO or iron supplementation, any bleeding episodes or blood transfusions, evident inflammation, diagnosis of an infectious disease, or diagnosis of malignancy in the 4 weeks prior to the commencement of the study. 

### 2.2. Samples

Blood specimens were collected during outpatient visits. Whole blood for the blood counts was collected by venipuncture into tubes containing trisodium EDTA. Serum samples were prepared immediately after the specimen was collected and stored at −80°C until the measurements were made. The serum was later thawed and used to measure serum iron, ferritin, total iron binding capacity (TIBC), transferrin, and TSAT. The TSAT level was calculated and the serum ferritin level measured as indicators of iron metabolism. The serum ferritin levels were measured with a Roche MODULAR Analytics analyzer. TSAT was calculated after measurement of the serum iron level and total iron binding capacity with a Hitachi automatic analyzer (model 7700, Nitoroso PSAP). Serum hepcidin levels were measured by surface-enhanced laser desorption ionization time-of-flight mass spectrometry (SELDI-TOF-MS), and IL-6 (interleukin-6) was measured by enzyme immunoassay.

### 2.3. Measurement of Reticulocyte Hemoglobin Content (RET-He)

Conventional erythrocyte parameters and RET-He were measured with a blood cell count analyzer (model XE-2100) and upgraded software (XE RET master, Sysmex). RET-He is measured by a fluorescent flow cytometry technique which in the reticulocyte channel, using a polymethine dye, and also measures the mean value of the forward light scatter intensity of mature erythrocytes and reticulocytes [8]. 

### 2.4. Statistical Analysis

Pearson's correlation coefficients were calculated by using the Dr SPSS II software program (SPSS Inc.). The significance of intergroup differences was tested by analysis of variance. *P* values < 0.05 were regarded as statistically significant. Two-tailed *P* < 0.05 were considered to indicate a statistically significant difference.

## 3. Results

### 3.1. Patient Profile

Table 1 summarizes the baseline data for each parameter analyzed. The mean age of the subjects as a whole was 64.0 ± 15.8 years old, and 13 of them (25%) had diabetes mellitus (DM). The subjects had been undergoing PD for 38.4 ± 35.2 (months). Anemia was present in 53%, and their mean Hb concentration and mean Ht were 9.9 ± 1.5 g/dL and 30.6 ± 4.6%, respectively. Their mean iron parameter values were above iron deficiency levels (TSAT; 32.3 ± 16.1%, ferritin 245.8 ± 169.2 ng/mL). The mean serum hepcidin level of the PD patients (*n* = 52) was 80.7 ± 59.4 ng/mL, and it was higher than the mean value reported for healthy subjects (10.8 ng/mL) [9]. The mean RET-He level of the PD patients was 32.3 ± 2.2 pg, and the normal mean that we previously reported in regular hemodialysis patients was 32.4 ± 4.0 pg [7].

### 3.2. Correlation between Serum Hepcidin Levels and Hb Concentrations

**Figure 1:**
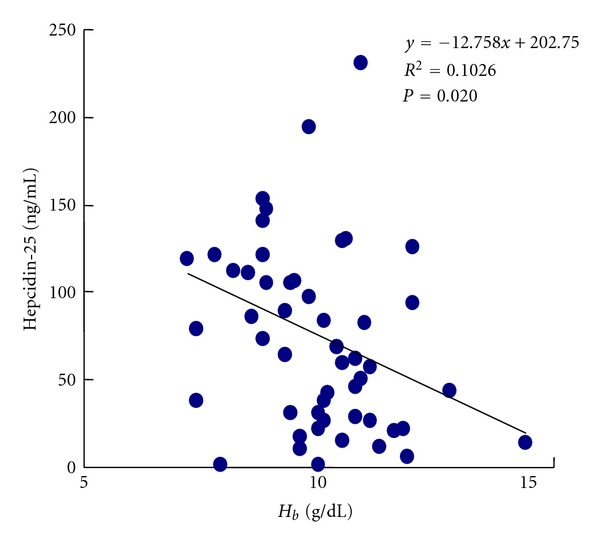
Correlation between serum hepcidin-25 levels and hemoglobin (Hb) concentrations.

**Figure 2:**
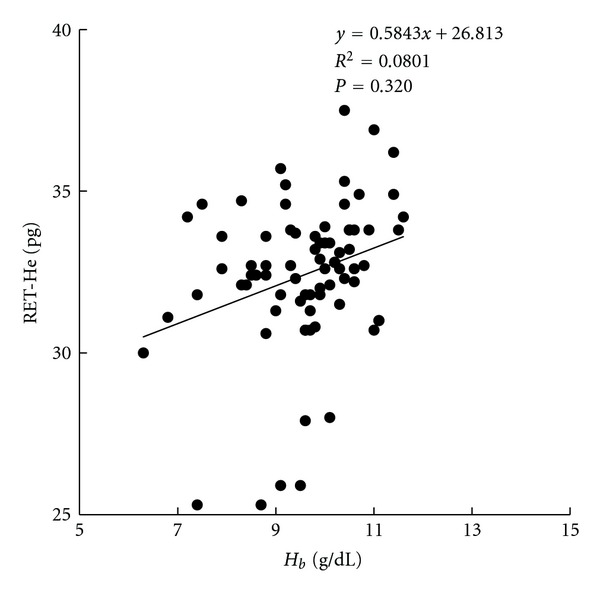
Correlation between serum RET-He and hemoglobin (Hb) concentrations.

As shown in Figure 1, the serum hepcidin levels were negatively correlated with the Hb concentrations. However, as to iron parameter, especially the direct iron marker of reticulocyte, there was no correlation between RET-He level and Hb level (Figure 2). 

### 3.3. Correlation between Iron Parameters and Serum Hepcidin Levels

**Figure 3:**
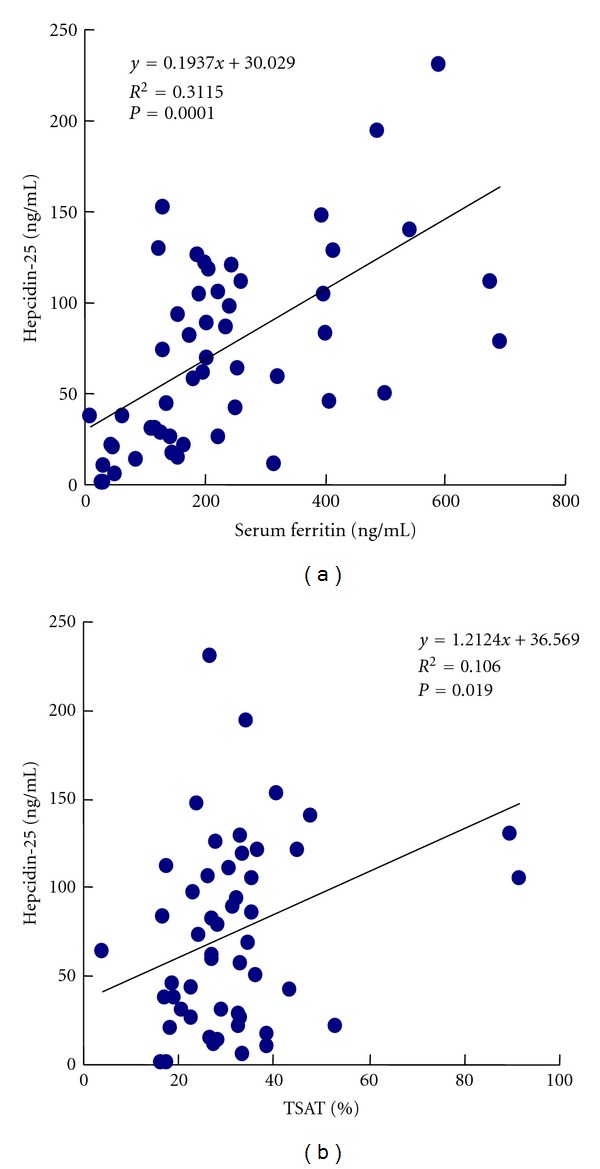
Correlation between serum hepcidin-25 levels and iron parameters. (a) Hepcidin and serum ferritin. (b) Hepcidin and TSAT.

Figure 3 shows the correlations between the serum hepcidin levels and iron marker levels of the PD patients. Significant correlations were found between the serum hepcidin levels and both the serum ferritin levels (*r* = 0.3115) and TSAT levels (*r* = 0.106), and the correlation coefficient indicated that the correlation between the serum hepcidin and the ferritin levels tended to be stronger. 

**Figure 4:**
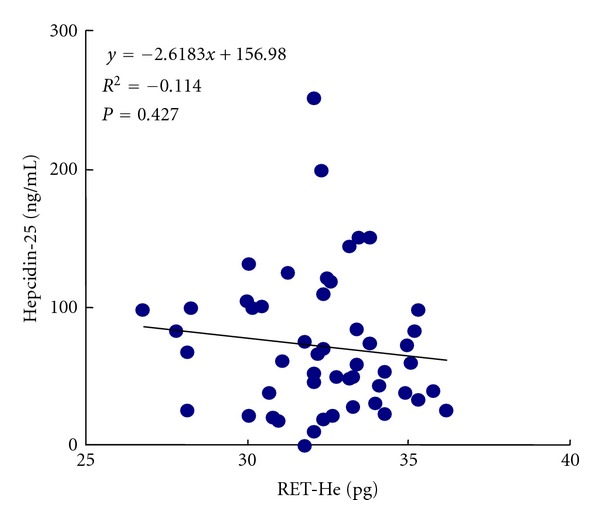
Correlation between serum hepcidin-25 levels and RET-He (reticulocyte hemoglobin content).

On the other hand, no correlation was found between their serum hepcidin levels and their RET-He levels (*r* = −0.114, *P* = 0.427), which are a direct measure of the iron content of newly produced erythrocytes, suggesting that hepcidin has no effect on the iron dynamics of reticulocytes (Figure 4).

### 3.4. Correlations between Inflammation Markers and Serum Hepcidin Levels

**Figure 5:**
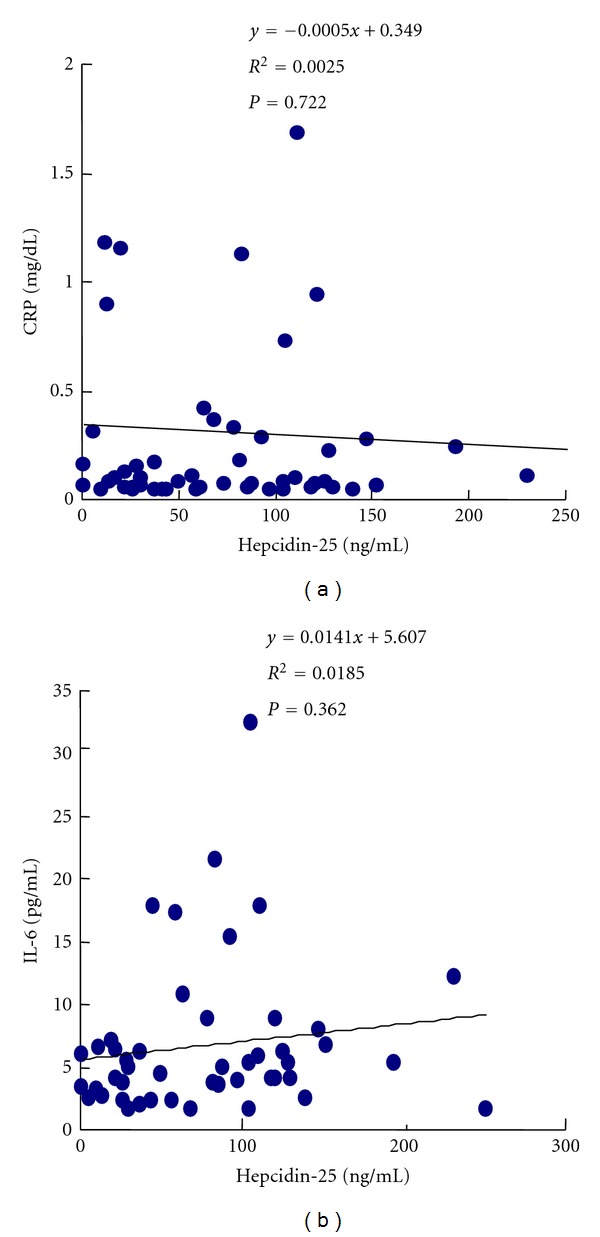
Correlation between serum hepcidin-25 levels and inflammation parameters. (a) Hepcidin and CRP. (b) Hepcidin and IL-6.

None of the patients was observed to have an infection or inflammation during the observation period, and their CRP and IL-6 values were low (CRP 0.3 ± 0.6 mg/dL, IL-6 5.6 ± 4.0 pg/mL) (Table 1). No significant correlations were found between their serum hepcidin levels and either their serum CRP levels (*r* = 0.0025, *P* = 0.722) or IL-6 levels (*r* = 0.0185, *P* = 0.362) (Figure 5). Since there were no significant correlations with the biomarkers of inflammation despite the fact that hepcidin expression is induced by IL-6, there is likely to be a threshold for stimulation of hepcidin expression by inflammation. 

### 3.5. Correlations between Serum IL-6 Levels and Iron Markers

**Figure 6:**
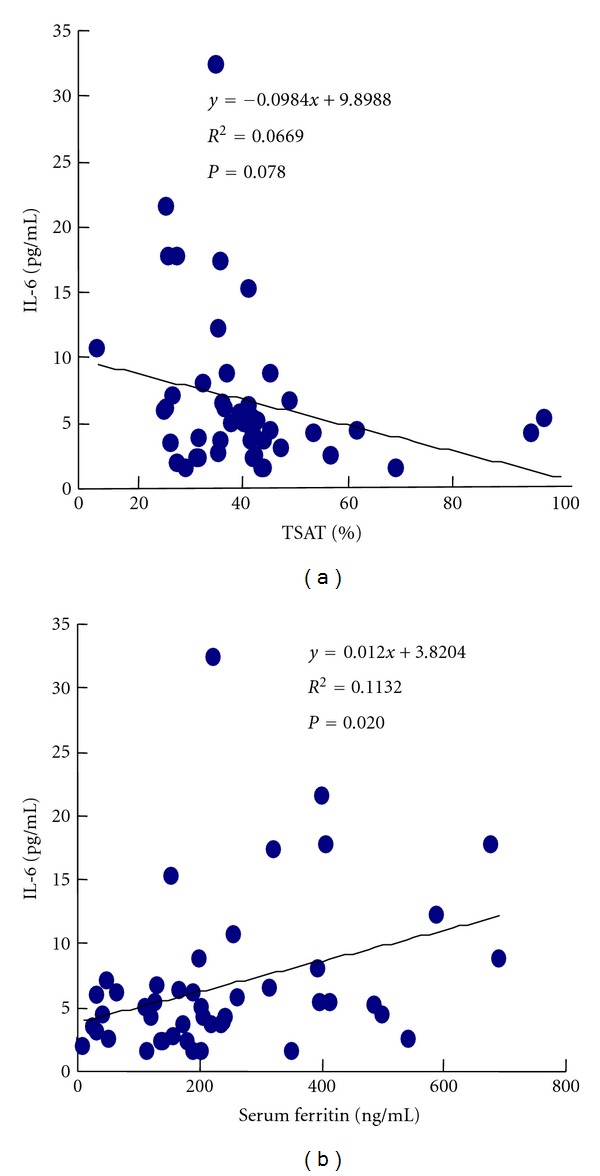
Correlation between serum IL-6 levels and iron parameters. (a) IL-6 and TSAT, (b) IL-6 and serum ferritin.

The serum IL-6 levels were not directly correlated with the values of the iron marker TSAT, but they were positively and significantly correlated with the serum ferritin levels (*r* = 0.1132, *P* < 0.020) (Figure 6).

## 4. Discussion

Hepcidin seems to have no direct effect on the iron status of erythrocytes, and expression of hepcidin is induced by a certain intensity of inflammatory stimulation. Long-acting rHuEPO stimulates erythropoiesis in the bone marrow, which utilizes iron, restores the Hb level, and then reduces the serum hepcidin level.

Measurements of serum hepcidin levels have not been reliable, because its protein structure is not specifically detected by enzyme immunoassay [10]. However, SELDI-TOF MS has made it possible to measure serum hepcidin levels [4]. There have been several reports regarding serum hepcidin levels in chronic kidney disease (CKD) [11–13]. Increased serum hepcidin levels were originally demonstrated in hemodialysis patients by the SELDI-TOF-MS method [4]. Although the absolute values of hepcidin have not been established, many reports have confirmed elevation of serum hepcidin levels in hemodialysis patients. The serum hepcidin levels of predialysis CKD patients tend to increase as their glomerular filtration rates are declined [14, 15], and they have been found to be altered by erythropoietin or intravenous iron administration. Several factors, including endogenous and exogenous erythropoietin, decreased erythropoiesis in the bone marrow, iron deficiency as a result of dietary restriction and overloading due to negative erythropoiesis, chronic stress conditions in CKD, and so forth, tend to modify hepcidin production and function. Thus, it is not easy to discriminate the pathophysiological condition of hepcidin status in CKD [16].

In this study, serum hepcidin levels were measured to clarify the role of hepcidin in PD patients, who had been receiving an injection of erythropoietin every month. The serum hepcidin level of PD patients may be affected by several factors, including continuous artificial fluid retention, chronic peritoneal irritation by the dialysate, and occult infection or inflammation in the peritoneum. There have been no reports regarding the serum hepcidin levels of PD patients measured by the SELDI-TOF-MS method. Malyszick L reported the serum hepcidin levels of PD patients measured as prohepcidin by enzyme assay. The hepcidin levels in that study were found to be correlated with residual kidney function, but the investigators measured prohepcidin and hepcidin by enzyme assays [17, 18]. In the present study, the mean serum hepcidin level of the PD patients was 80.7 ± 59.4 ng/mL, which was higher than the previously reported level measured by the SELDI-TOF-MS method in hemodialysis patients.

Hepcidin is thought to be the major regulator of dietary iron absorption and cellular iron release, and it exerts its regulatory function by counteracting the function of ferroportin, the major cellular iron exporter in the various cells membrane [19]. Hepcidin induces the internalization and degradation of ferroportin [20], resulting in increasing intracellular iron stores, decreased dietary iron absorption, and decreased circulating iron levels. Hepcidin controls the entry of iron into the plasma mediated by ferroportin, and there is a crosstalk between plasma iron saturation or iron stores and plasma hepcidin level in physiological state. Iron stores and circulating transferrin bound iron provide distinct signals that affect hepcidin synthesis in hepatocytes [21, 22], resulting in the positive correlation between serum iron biomarkers and hepcidin levels.

 In addition, several physiologic and pathologic processes regulate hepcidin synthesis. Hepcidin levels are likely to be regulated by several independent mechanisms, as previously reviewed [23]. Conditions in which demand for circulating iron is increased induce a decrease in hepatocellular hepcidin synthesis, and a decrease in the serum hepcidin level results in the release of stored iron and an increase in dietary iron absorption. Such conditions include iron deficiency, hypoxia, and condition in which erythropoietic activity is increased. Particularly, high erythropoietic activity demands sufficient iron supply suppresses hepcidin synthesis. Thus, there is negative correlation between Hb concentration and hepcidin levels as shown in Figure 1. 

In this study, the similar relation among hepcidin levels, Hb concentration, and iron parameters except for RET-He could be observed. PD patients' serum hepcidin levels were significantly positively correlated with their serum ferritin and TSAT levels, but no correlations were found between their serum hepcidin levels and RET-He levels, suggesting that hepcidin has no effect on the iron dynamics of reticulocytes. Stimulation of erythropoiesis by ESA therapy increases the demand for instantly available iron, which often proves insufficient even in patients whose whole body iron store is not significantly depleted [24, 25]. Absolute iron deficiency in HD patients has been defined on the basis of TSAT and serum ferritin levels, whereas functional iron deficiency results when there is a need for a greater amount of iron to support erythropoiesis than can be supplied. Thus, the conventional methods of estimating iron stores, such as serum ferritin and TSAT measurements, are inadequate to evaluate functional iron deficiency. A strong correlation between serum ferritin and TSAT levels and serum hepcidin levels has been confirmed, but there is no information about the relation between hepcidin and reticulocyte hemoglobin. No correlation was found between the serum hepcidin levels and reticulocyte hemoglobin levels in this study, suggesting that hepcidin does not directly regulate iron metabolism in newly produced erythrocytes.

The primary mediator of inflammation seems to be IL-6, which causes the signal transducer and activator of transcription-3 to bind to the hepcidin promoter, increasing its activity [26]. Previous studies have shown markedly increased serum hepcidin levels in humans with chronic infections and severe inflammatory diseases, suggesting that elevated serum hepcidin levels play a key role in the anemia of inflammation and reticuloendothelial blockade [27]. Correlations between serum hepcidin levels and serum levels of inflammatory markers, including IL-6, IL-1, and high sensitive CRP, have been found in several studies [4, 15]. However, several studies have not necessarily shown the relationship between serum hepcidin levels and the levels of these inflammatory markers [11, 12]. Since low levels of CRP and IL-6, biomarkers of inflammation, were not correlated with the serum hepcidin levels, there is likely to be a threshold for stimulation of hepcidin induction by inflammation.

## 5. Conclusion 

SELDI-TOF-MS measurements showed that the PD patients in this study had high serum hepcidin levels, nevertheless in case of peritonitis or in high levels of biomarker indicating inflammation. Good correlations were found between the PD patients' serum hepcidin levels and both their TSAT ferritin levels, the same as reported previously, but hepcidin was found to have no direct effect on erythrocyte iron status. In inflammatory conditions, the primary mediator seems to be IL-6 levels and induces hepcidin expression; there has been no definite causal relationship in the regular status of PD patients.

## Figures and Tables

**Table 1:** The profile of the patients.

Number	52
Sex F/M	22/30
DM *n* (%)	13 (25)
Age (year)	64.0 ± 15.8
Duration of PD (month)	38.4 ± 35.2
BUN (mg/dL)	54.5 ± 13.5
Cr (mg/dL)	9.1 ± 3.9
TP (g/dL)	6.2 ± 0.6
Kt/V	2.1 ± 0.5
Weekly CCr	75.6 ± 29.8
Urine volume (mL/day)	881.1 ± 559.4
Anemia *n* (%)	28 (53.8)
Iron deficiency *n* (%)	4 (7.7)
Hb (g/dL)	9.9 ± 1.5
Ht (%)	30.6 ± 4.6
Fe (*μ*g/dL)	84.1 ± 36.7
TIBC (*μ*g/dL)	287.0 ± 155.0
TSAT (%)	32.3 ± 16.1
Ferritin (ng/mL)	245.8 ± 169.2
Ret (%)	7.7 ± 4.5
RET-He (pg)	32.3 ± 2.2
Hepcidin-25 (ng/mL)	80.7 ± 59.4
CRP (mg/dL)	0.3 ± 0.6
IL-6 (pg/mL)	5.6 ± 4.0
ESA use *n* (%)	48 (92.3)
I Epoetin beta *n* (%)	16 (30.8)
I Epoetin beta (U/month)	13875 ± 6469
I Dalbepoetin alpha *n* (%)	32 (61.5)
I Dalbepoetin alpha (*μ*g/month)	133 ± 77
